# Development and validation of a 10‐gene prognostic signature for acute myeloid leukaemia

**DOI:** 10.1111/jcmm.15109

**Published:** 2020-03-09

**Authors:** Zuyi Yang, Jun Shang, Ning Li, Liang Zhang, Tingting Tang, Guoyan Tian, Xiaohui Chen

**Affiliations:** ^1^ Department of Hematology and Oncology The Affiliated Hospital of Hangzhou Normal University Hangzhou China; ^2^ School of Life Sciences Fudan University Shanghai China

**Keywords:** acute myeloid leukaemia, gene expression profiling, nomogram, prognosis, signature

## Abstract

Acute myeloid leukaemia (AML) is the most common type of adult acute leukaemia and has a poor prognosis. Thus, optimal risk stratification is of greatest importance for reasonable choice of treatment and prognostic evaluation. For our study, a total of 1707 samples of AML patients from three public databases were divided into meta‐training, meta‐testing and validation sets. The meta‐training set was used to build risk prediction model, and the other four data sets were employed for validation. By log‐rank test and univariate COX regression analysis as well as LASSO‐COX, AML patients were divided into high‐risk and low‐risk groups based on AML risk score (AMLRS) which was constituted by 10 survival‐related genes. In meta‐training, meta‐testing and validation sets, the patient in the low‐risk group all had a significantly longer OS (overall survival) than those in the high‐risk group (*P* < .001), and the area under ROC curve (AUC) by time‐dependent ROC was 0.5854‐0.7905 for 1 year, 0.6652‐0.8066 for 3 years and 0.6622‐0.8034 for 5 years. Multivariate COX regression analysis indicated that AMLRS was an independent prognostic factor in four data sets. Nomogram combining the AMLRS and two clinical parameters performed well in predicting 1‐year, 3‐year and 5‐year OS. Finally, we created a web‐based prognostic model to predict the prognosis of AML patients (https://tcgi.shinyapps.io/amlrs_nomogram/).

## 1 INTRODUCTION

Acute myeloid leukaemia (AML) is haematologic malignancy with high heterogeneity, characterized by uncontrolled proliferation of myeloid progenitor cells gradually replacing the normal haematopoietic function of bone marrow. With the continuous exploration and research at the cellular and molecular level on the pathogenesis of AML, the choice of novel treatment modalities has surged over the past few years, including targeted small‐molecule inhibitors, antibody‐drug conjugate, tumour‐targeted immunotherapy and so on.^1^, ^2^ The prognosis of majority of young AML patients has improved, and most patients have access to complete remission. However, more than half of young adult patients and approximately 90% of older patients still die of their diseases.^3^ Hence, a reliable prognostic stratification system which can be applied to clinical risk evaluation is of high importance for the choice of therapy and follow‐up in AML patients.

Whether it is an established classification system, such as the French‐American‐British (FAB) classification system in 1976,^4^ World Health Organization (WHO) classification in 2008^5^ and 2016^6^ incorporating genetic information, or prognostic factors, for instance, clinical factors including mounting age and poor performance status,^7^ cytogenetic changes^8^ and gene mutation,^9^ all have their downsides for risk stratification, such as the insufficiency of generalization capacity, the uncertainty in the accuracy of prediction. Hence, recently increasing sight has turned to studies on risk prediction models by prognostic signature based on multiple gene integration for different types of tumours, especially in solid tumours. In this study, we aimed to construct a prognostic signature based on gene expression profile from public database and validate its stability and forecasting performance, as well as establish a clinically applied nomogram for AML risk stratification.

## 2 MATERIALS AND METHODS

### 2.1 Data sources

**Figure 1:**
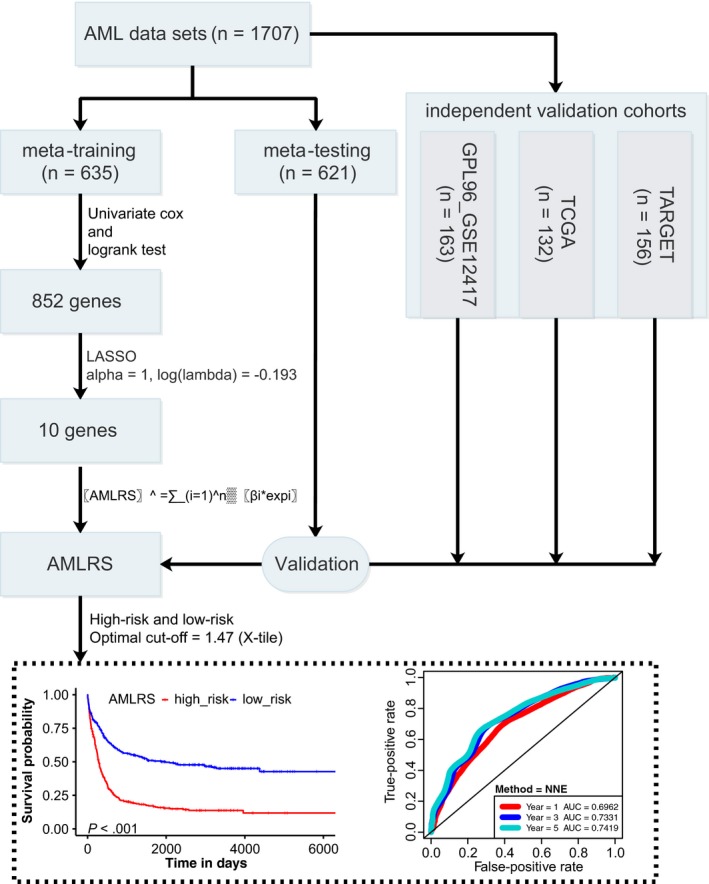
The workflow of the study. A total of five data sets including 1707 AML patient samples were analysed. Survival‐related genes were screened in meta‐training set and eventually used to build AMLRS. Meta‐testing and three independent validation cohorts were utilized for validation. An optimal cut‐off was identified by X‐title (1.47) and divided patients into high‐risk and low‐risk groups. In meta‐training set, the patient in the low‐risk group had a significantly longer OS than those in the high‐risk group (*P* < .001), and the AUC was 0.6962 for 1 year, 0.7331 for 3 years and 0.7419 for 5 years, respectively. AML, acute myeloid leukaemia; AMLRS, AML risk score; OS, overall survival

We retrospectively gathered the gene expression profile data and corresponding clinical information of AML patients from three cancer public data sets. A total of eight cohorts were included in the study, including six cohorts from the Gene Expression Omnibus (GEO) database, one from The Cancer Genome Atlas (TCGA) database and one from Therapeutically Available Research to Generate Effective Treatments (TARGET) database. After deleting those data without survival and expression information, a total of 1707 AML samples were ultimately enrolled in our study, including 1419 GEO AML samples, 132 TCGA AML samples and 156 TARGET AML samples. Detailed information about the data sets was described in Table S1. The workflow was drawn in Figure 1.

### 2.2 Data processing

**Figure 2:**
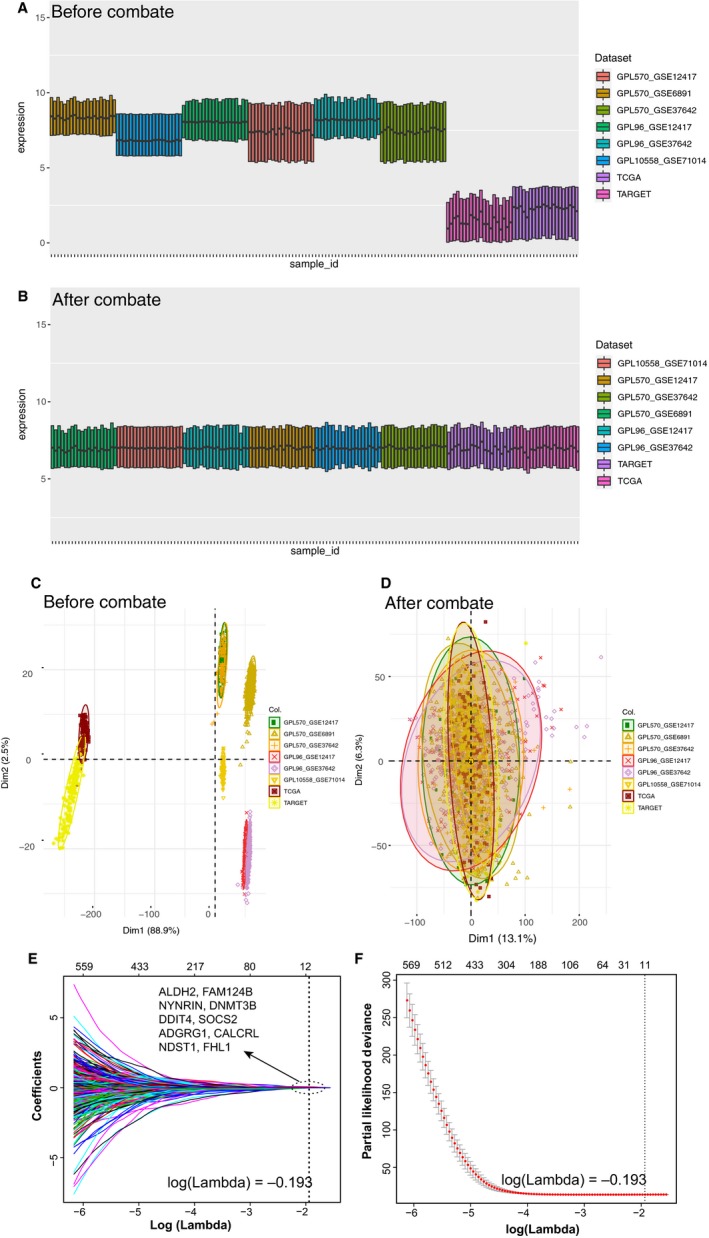
Comparison before and after batch effect elimination on different cohorts. The boxplot shown gene expression of eight cohorts, each of which contained 20 random samples (A) before and (B) after combat. The PCA of eight cohorts (C) before and (D) after combat. Prognosis‐related gene selection in the LASSO‐COX regression. (E) LASSO coefficient values of the 10 prognosis‐related genes in training cohort. The dotted vertical line at the log(Lambda) = −0.193. (F) L1‐penalty of LASSO‐COX regression. The dotted vertical lines at optimal log(Lambda) value

The gene expression data and corresponding clinical information of http://www.ncbi.nlm.nih.gov/geo/query/acc.cgi?acc=GSE12417, http://www.ncbi.nlm.nih.gov/geo/query/acc.cgi?acc=GSE37642, http://www.ncbi.nlm.nih.gov/geo/query/acc.cgi?acc=GSE6891 and http://www.ncbi.nlm.nih.gov/geo/query/acc.cgi?acc=GSE71014 data sets were collected from GEO data sets (https://www.ncbi.nlm.nih.gov/geo/). Affymetrix microarray data of http://www.ncbi.nlm.nih.gov/geo/query/acc.cgi?acc=GSE12417, http://www.ncbi.nlm.nih.gov/geo/query/acc.cgi?acc=GSE37642 and http://www.ncbi.nlm.nih.gov/geo/query/acc.cgi?acc=GSE6891 data sets were downloaded in the form of CEL file and adjusted by Robust Multichip Average (RMA) normalization method (R package affy, V1.60.0). Because cohorts of http://www.ncbi.nlm.nih.gov/geo/query/acc.cgi?acc=GSE12417 and http://www.ncbi.nlm.nih.gov/geo/query/acc.cgi?acc=GSE37642 hybridized on Affymetrix U133B had repeated samples and small number of gene probe, we removed cohorts of http://www.ncbi.nlm.nih.gov/geo/query/acc.cgi?acc=GSE12417 and http://www.ncbi.nlm.nih.gov/geo/query/acc.cgi?acc=GSE37642 hybridized on Affymetrix U133B. For http://www.ncbi.nlm.nih.gov/geo/query/acc.cgi?acc=GSE71014, normalized expression data were downloaded. The expression data and corresponding clinical information of TCGA and TARGET data sets were downloaded from UCSC (https://xenabrowser.net/hub/), and logarithmic transformed was done in all gene expression. All data were adjusted with ComBat method (R package sva, V3.30.0) to eliminate the batch effect between different data sets (Figure 2A‐D). We merged the GPL570‐http://www.ncbi.nlm.nih.gov/geo/query/acc.cgi?acc=GSE6891, GPL570‐http://www.ncbi.nlm.nih.gov/geo/query/acc.cgi?acc=GSE37642, GPL96‐http://www.ncbi.nlm.nih.gov/geo/query/acc.cgi?acc=GSE37642 and GPL570‐http://www.ncbi.nlm.nih.gov/geo/query/acc.cgi?acc=GSE124177 data sets into a meta‐data set and randomly divided this data set into meta‐training set (n = 635) and meta‐testing set (n = 621) in a 1‐to‐1 ratio. Meanwhile, GPL96‐http://www.ncbi.nlm.nih.gov/geo/query/acc.cgi?acc=GSE12417 (n = 163), TCGA (n = 132) and TARGET (n = 156) were utilized as independent cohorts for validation of our prognostic prediction model.

### 2.3 Construction and validation of the prognostic signature for AML

The log‐rank test and univariate COX regression analysis were used to screen survival‐related genes in the meta‐training set. 1000 time iterations were carried out by using LASSO‐COX (R package glmnet, v2.0‐16),^10^ to identify the most stable gene set. AML risk score (AMLRS) was calculated by using the linear weighted method of covariates based on COX regression and gene expression value of target genes, and each patient got an AMLRS, the formulate as shown below 11, 12:

$$\text{AMLRS}=\sum_{i=1}^{N}\beta i*\exp\;i$$

The optimal cut‐off value identified by X‐title method was utilized to divide patients into the low‐risk and high‐risk groups.^13^ Time‐dependent receiver operating characteristic (ROC) and Kaplan‐Meier survival analysis were employed to assess the prognostic predictive power of AMLRS (R package, survivalROC, v1.0.3). The meta‐testing, GPL96‐http://www.ncbi.nlm.nih.gov/geo/query/acc.cgi?acc=GSE12417, TCGA and TARGET data sets were carried out to validate the stability of AMLRS.

A predictive nomogram was performed to build clinically applicable scale plate (R package, rms, v5.1‐2). Calibration plots were carried out to evaluate the forecasting performance of the nomogram (R package, rms, v5.1‐2). Online prognostic tool was built with shiny (R package, shiny, v1.2.0).

### 2.4 Statistical analysis

The characteristics of gene expression and corresponding clinical information were displayed with heatmap (R package, ComplexHeatmap, v1.18.1).^14^ The principal component analysis (PCA) was conducted for clustering gene expression data (R package, gmodels, v 2.18.1). The chi‐square test was used to compare the statistical difference in categorical variables, and two‐tailed Student's *t* test was used for quantitative variables (SPSS version 19.0; IBM Corporation). The violin plot was performed with ggplot2 (R package, ggplot2, v3.0.0). Univariate and multivariate COX regression analyses were performed to evaluate the association between variables and overall survival (OS) (R package, survival, v2.42.6). Kaplan‐Meier survival analysis was carried out to compare the difference in survival among groups (R package, survminer, v0.4.3). A *P* value < .05 was considered as statistical significance.

## 3 RESULTS

### 3.1 Patient characteristics

In five data sets, a total of 1707 AML patients were analysed, including 390 males (22.8%), 355 females (20.8%) and 962 of unknown sex (56.4%). Except for TARGET data set, which consisted of paediatric and adolescent AML patients, the majority of patients in other data sets were adult AML patients, ranging from 15 to 88. In patients with known data, white blood cells (WBC) > 10 (12.5%) and platelet (PLT) counts < 100 (6.2%) patients comprised the majority, and M1 (19%), M2 (24.4%), M4 (19.2%) and M5 (13.8%) subtype account for a large proportion in different FAB subtype patients. In patients of known cytogenetic risk stratification and cytogenetic abnormalities, intermediate‐risk group and normal karyotype group were the most common subtypes, accounting for 23.8% and 30.8, respectively. The median follow‐up times for the five data sets (meta‐training, meta‐testing, GPL96‐http://www.ncbi.nlm.nih.gov/geo/query/acc.cgi?acc=GSE12417, TCGA and TARGET) were 425, 459.3, 280, 366 and 1348.5 days, respectively. The characteristics of data sets were displayed in Table S2.

### 3.2 Construction of the prognostic signature

In our study, a total of 12 272 genes were investigated. Screening by log‐rank test and univariate COX regression analysis, 852 genes were found as survival‐related gene. To reduce the risk of overfitting after initial screening, a LASSO‐COX was used. After 1000 iterations, a 10‐gene signature was considered as the most stable gene set in the meta‐training set (alpha = 1, Log (Lambda) = 0.193) (Figure 2E‐F), including ALDH2, FAM124B, NYNRIN, DNMT3B, DDIT4, SOCS2, ADGRG1, CALCRL, NDST1 and FHL1 (the detail information of screen was presented in Table S3). The frequency of this gene signature was up to 224 times and was the highest frequency in different gene signatures (Figure S1). Using the linear weighting for the 10 genes, a formula of AMLRS was constructed, integrating the gene expression value and the coefficients derived from multivariable COX regression. The AMLRS of each patient in our study was calculated, and the formula was exhibited below:

$$\text{AMLRS}=\text{ALDH}2\left(\text{expression}\;\text{level}\right)*0.0152+\text{FAM}124B*0.017+\text{NYNRIN}*0.007+\text{DNMT}3B*0.021+\text{DDIT}4*0.015+\text{SOCS}2*0.0197+\text{ADGRG}1*0.039+\text{CALCRL}*0.072+\text{NDST}1*\left(-0.015\right)+\text{FHL}1*0.010.$$

**Figure 3:**
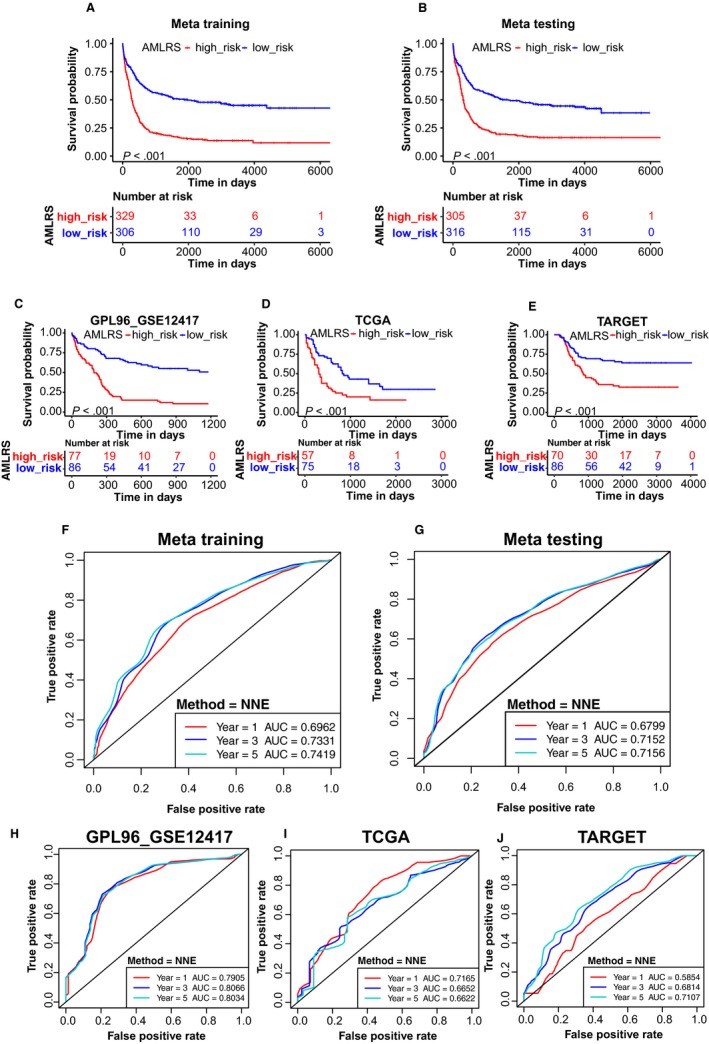
The Kaplan‐Meier survival analysis in five data sets. A, Meta‐training data set. B, meta‐testing data set. C, GPL96_GSE12417 data set, D, TCGA data set. E, TARGET data set. In five data sets, the patient in the low‐risk group all had a significantly longer OS than those in the high‐risk group (*P* < .001). The time‐dependent ROC in five data sets. F, In meta‐training data set, the AUC was 0.6962 for 1 y, 0.7331 for 3 y and 0.7419 for 5 y, respectively. G, In meta‐testing data set, the AUC was 0.6799 for 1 y, 0.7152 for 3 y and 0.7156 for 5 y, respectively. H, In GPL96_GSE12417 data set, the AUC was 0.7905 for 1 y, 0.8066 for 3 y and 0.8034 for 5 y, respectively. I, In TCGA data set, the AUC was 0.7165 for 1 y, 0.6652 for 3 y and 0.6622 for 5 y, respectively. J, In TARGET data set, the AUC was 0.5854 for 1 y, 0.6814 for 3 y and 0.7107 for 5 y, respectively

**Figure 4:**
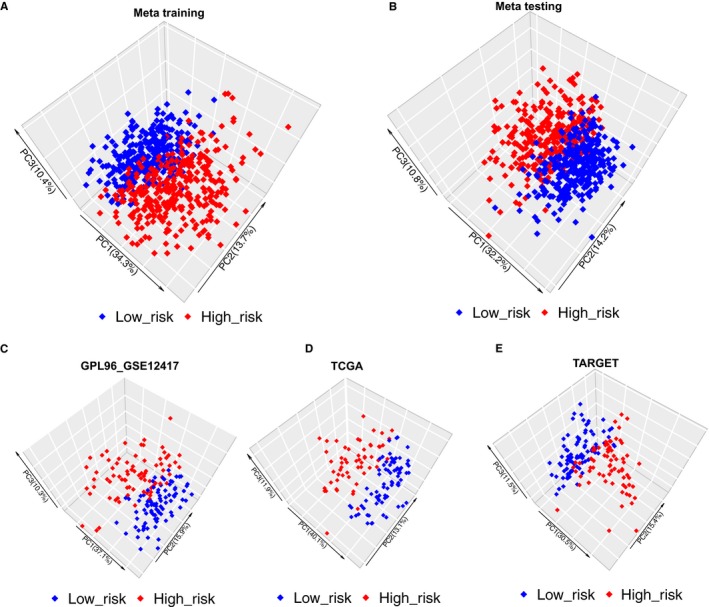
The PCA of the low‐risk and high‐risk groups based on 10 survival‐related gene expression. A, Meta‐training data set. B, Meta‐testing data set. C, GPL96_GSE12417 data set. D, TCGA data set. E, TARGET data set

By X‐title, an optimal cut‐off value (1.47) was calculated to divide patients into low‐risk and high‐risk groups (Figure S2). In meta‐training set, the patient in the low‐risk group had a significantly longer OS than those in the high‐risk group (*P* < .001) (Figure 3A), and the area under ROC curve (AUC) by time‐dependent ROC was 0.6962 for 1 year, 0.7331 for 3 years, and 0.7419 for 5 years, respectively (Figure 3F). The 1, 3 and 5‐year survival rates in the high‐risk vs low‐risk patient were 41.6% vs 71.6%, 19.8% vs 55.7% and 15.8% vs 50.5%, respectively. According to PCA, the expression of 10 genes could distinguish well the low‐risk group from the high‐risk group in meta‐training set (Figure 4A). The results obtained from the foregoing analysis demonstrated that the prognostic signature had a good prognostic performance.

### 3.3 Validation of the prognostic signature

To evaluate the generalization capacity of the prognostic signature, we validated the meta‐testing set and three independent validation data sets, including GPL96‐http://www.ncbi.nlm.nih.gov/geo/query/acc.cgi?acc=GSE12417, TCGA and TARGET data sets. In four validation data sets, the patients in the low‐risk group all had a significantly longer OS than those in the high‐risk group (*P* < .001; Figure 3B‐E). Meanwhile, the time‐dependent ROC was drawn in four validation data sets (Figure 3G‐J). The AUC in meta‐testing data set was 0.6799 for 1 year, 0.7152 for 3 years and 0.7156 for 5 years, respectively. The 1, 3 and 5‐year survival rates in the high‐risk vs low‐risk patient were 42.7% vs 70.8%, 22.2% vs 55.3% and 19.0% vs 49.3%, respectively. The AUC in GPL96‐http://www.ncbi.nlm.nih.gov/geo/query/acc.cgi?acc=GSE12417 data set was 0.7905 for 1 year, 0.8066 for 3 years and 0.8034 for 5 years, respectively. The 1 and 3‐year survival rates in the high‐risk vs low‐risk patient were 19.7% vs 67.9% and 10.6% vs 52.9%, respectively. The AUC in TCGA data set was 0.7165 for 1 year, 0.6652 for 3 years, and 0.6622 for 5 years, respectively. The 1, 3, and 5‐year survival rates in the high‐risk vs low‐risk patient were 41.5% vs 73.1%, 20.0% vs 42.9% and 16.0% vs 29.7%, respectively. The AUC in TARGET data set was 0.5854 for 1 year, 0.6814 for 3 years and 0.7107 for 5 years, respectively. The 1, 3 and 5‐year survival rates in the high‐risk vs low‐risk patient were 85.5% vs 90.7%, 43.3% vs 69.2% and 34.2% vs 65.3%, respectively. According to PCA, the expression of 10 genes could distinguish well the low‐risk group from the high‐risk group in testing and validation data sets (Figure 4B‐E).

### 3.4 Subgroup analysis fusing with clinical characteristics

**Figure 5:**
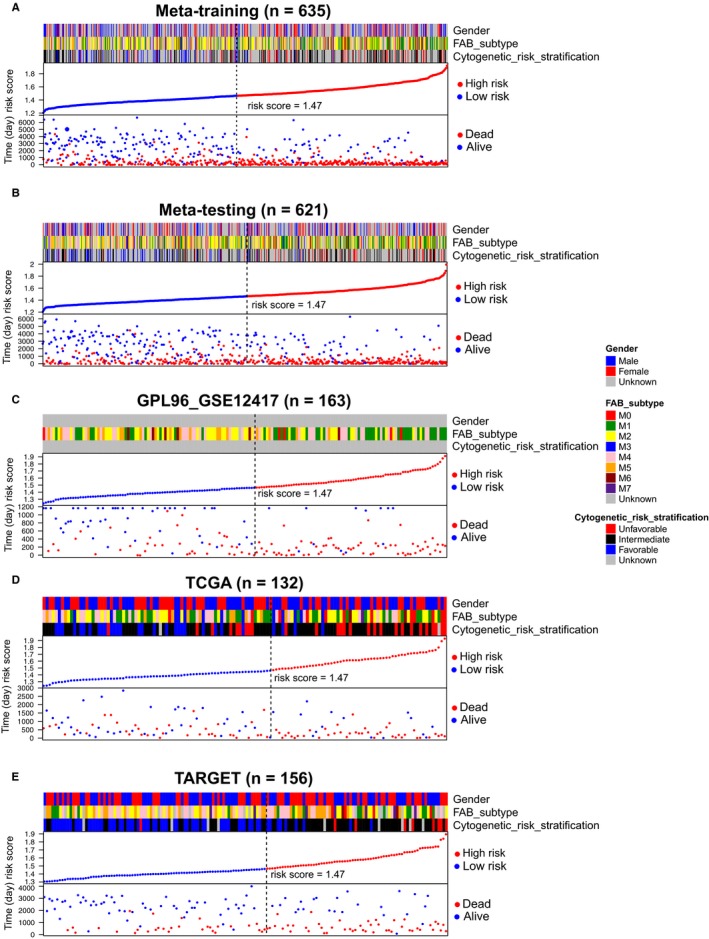
The heat map of risk score distribution, survival status and clinical parameters in five data sets. Each column represented a patient, and each row represented gender, FAB subtype, cytogenetic risk stratification, risk score stratification and survival status and corresponding survival time, respectively. All patients were ranked by risk score. A, Meta‐training data set. B, Meta‐testing data set. C, GPL96_GSE12417 data set. D, TCGA data set. E, TARGET data set. FAB, French‐American‐British

We created heatmap in five data sets integrating AMLRS, survival statue and clinical characteristics, containing gender, FAB subtype and cytogenetic risk stratification (Figure 5), and the gene expression value of 10 genes was displayed in Figure S3. Patients with high AMLRS scores were more distributed in the unfavourable karyotype group, M0 and M1 subtypes, while patients with low AMLRS scores were more distributed in the favourable karyotype group, M3, M4 and M5 subtypes (Figure S4). Meantime, using time‐dependent ROC we compared AMLRS and cytogenetic risk stratification, although the ROC curve results suggested that AMLRS model was better, the cytogenetic risk stratification also performed very well (Figure S5). In 1707 AML patients, patients in M3 subtype and favourable karyotype group had longer OS (*P* < .001), while patients in M0 subtype and unfavourable karyotype group had shorter OS (*P* < .001; Figure S6A,C). Patients in unfavourable karyotype group had the highest risk score, followed by the intermediate karyotype group, and favourable karyotype group had the lowest one (*P* < .001; Figure S6B). In FAB subtype, patients in M0 and M3 subtype had the highest and lowest risk score, respectively, while risk score for other types of patients was between the two groups (*P* < .001; Figure S6D). In cytogenetic abnormality, inv (16), *t*(8; 21) and *t*(15; 17) were more likely to be observed in the low‐risk group, while −5/7(*q*), +8 and complex cytogenetic abnormalities were more likely to be observed in the high‐risk group, that was consistent with genomic risk stratification of AML.^15^ Detailed information about clinical characteristics was described in Table 1.

**Table 1:** Comparison of clinical characteristics between the low‐risk and high‐risk groups in 5 data sets

Factors	Meta‐training n (%)			Meta‐testing n (%)			GPL96_GSE12417 n (%)			TCGA n (%)			TARGET n (%)		
	Low risk	High risk	*P*	Low risk	High risk	*P*	Low risk	High risk	*P*	Low risk	High risk	*P*	Low risk	High risk	*P*
Total	306	329		316	305		86	77		75	57		86	70	
Gender															
Male	66 (21.6)	61(18.5)	ns	54 (17.1)	59 (19.3)	ns	0	0	ns	37 (49.3)	34 (59.6)	ns	47 (54.7)	32 (45.7)	ns
Female	50 (16.3)	65 (19.8)		58 (18.4)	44 (14.4)		0	0		38 (50.7)	23 (40.4)		39 (45.3)	38 (54.3)	
Unknown	190 (62.1)	203 (61.7)		204 (64.6)	202 (66.2)		86	77		0	0		0	0	
Age (median)	48^a^	53^a^	***	49^b^	55^b^	*	55	61	ns	51	61	*	3291.5^c^	3348.5^c^	ns
Race															
White	0	0		0	0		0	0		68(90.7)	50 (87.7)	ns	65 (75.6)	56 (80)	ns
Black or African American	0	0		0	0		0	0		4 (5.3)	7 (12.3)		11 (12.8)	5 (7.1)	
Asian	0	0		0	0		0	0		1 (1.3)	0		2 (2.3)	1 (1.4)	
Other	0	0		0	0		0	0		0	0		6 (7)	4 (5.7)	
Unknown	306	329		316	305		86	77		2 (2.7)	0		2 (2.3)	4 (5.7)	
WBC															
<4	0	0	–	0	0	–	0	0	–	10 (13.3)	20 (35.1)	*	2 (2.3)	5 (7.1)	ns
4‐10	0	0		0	0		0	0		14 (18.7)	9 (15.8)		8 (9.3)	5 (7.1)	
>10	0	0		0	0		0	0		50 (66.7)	28 (49.1)		76 (88.4)	60 (85.7)	
Unknown	0	0		0	0		0	0		1 (1.3)	0		0	0	
HB															
<12	0	0	–	0	0	–	0	0	–	71 (94.7)	47 (82.5)	ns	0	0	–
≥12	0	0		0	0		0	0		4 (5.3)	9 (15.8)		0	0	
Unknown	0	0		0	0		0	0		0	1 (1.8)		0	0	
PLT															
<100	0	0	–	0	0	–	0	0	–	63 (84)	43 (75.4)	ns	0	0	–
≥100	0	0		0	0		0	0		12 (16)	14 (24.6)		0	0	
BM blast cells	0	0		0	0		0	0		39.5^d^	48.5^d^	ns	74.8	73.1^e^	ns
FAB subtype															
M0	2 (0.7)	17 (5.2)	***	3 (0.9)	17 (5.6)	***	1 (1.2)	4 (5.2)	***	2 (2.7)	10 (17.5)	*	0	4 (5.7)	*
M1	39 (12.7)	78 (23.7)		41 (13)	72 (23.6)		13 (15.1)	32 (41.6)		19 (25.3)	13 (22.8)		6 (7)	11 (15.7)	
M2	74 (24.2)	74 (22.5)		80 (25.3)	74 (24.3)		29 (33.7)	16 (20.8)		16 (21.3)	16 (28.1)		25 (29.1)	12 (17.1)	
M3	20 (6.5)	6 (1.8)		14 (4.4)	10 (3.3)		0	0		9 (12)	5 (8.8)		0	0	
M4	60 (19.6)	47 (14.3)		69 (21.8)	40 (13.1)		22 (25.6)	20 (26)		19 (25.3)	8 (14)		27 (31.4)	16 (22.9)	
M5	53 (17.3)	38 (11.6)		56 (17.7)	28 (9.2)		15 (17.4)	4 (5.2)		10 (13.3)	2 (3.5)		17 (19.8)	13 (18.6)	
M6	7 (2.3)	7 (2.1)		10 (3.2)	7 (2.3)		5 (5.8)	1 (1.3)		0	2 (3.5)		0	2 (2.9)	
M7	0	2 (0.6)		0	1 (0.3)		0	0		0	1 (1.8)		2 (2.3)	5 (7.1)	
Unknown	51 (16.7)	60 (18.2)		43 (13.6)	56 (18.4)		1 (1.2)	0		0	0		9 (10.5)	7 (10)	
Cytogenetic risk stratification			***			***			‐			***			***
Favourable	41 (13.4)	5 (1.5)		47 (14.9)	4 (1.3)		0	0		26 (34.7)	4 (7)		55 (64)	9 (12.9)	
Intermediate	57 (18.6)	83 (25.2)		57 (18)	62 (20.3)		0	0		39 (52)	34 (59.6)		26 (30.2)	48 (68.6)	
Unfavourable	16 (5.2)	34 (10.3)		8 (2.5)	33 (10.8)		0	0		9 (12)	18 (31.6)		1 (1.2)	8 (11.4)	
Unknown	192 (62.7)	207 (62.9)		204 (64.6)	206 (67.5)		86	77		1 (1.3)	1 (1.8)		4 (4.7)	5 (7.1)	
Cytogenetic abnormality															
Normal	71 (23.2)	70 (21.3)	***	71 (22.5)	54 (17.7)	***	86	77	–	33 (44)	29 (50.9)	*	19 (22.1)	16 (22.9)	***
‐5/7 (q)	1 (0.3)	15 (4.6)		1 (0.3)	13 (4.3)		0	0		5 (6.7)	11 (19.3)		0	0	
+8	5 (1.6)	6 (1.8)		3 (0.9)	6 (2)		0	0		4 (5.3)	7 (12.3)		1 (1.2)	2 (2.9)	
11q23	2 (0.7)	3 (0.9)		1 (0.3)	4 (1.3)		0	0		0	0		0	0	
inv(16)	17 (5.6)	0		16 (5.1)	0		0	0		8 (10.7)	0		19 (22.1)	2 (2.9)	
*t*(9; 22)	0	1 (0.3)		0	1 (0.3)		0	0		0	0		0	0	
*t*(15; 17)	7 (2.3)	2 (0.6)		9 (2.8)	3 (1)		0	0		9 (12)	4 (7)		0	0	
*t*(8; 21)	14 (4.6)	2 (0.6)		18 (5.7)	1 (0.3)		0	0		7 (9.3)	0		5 (5.8)	0	
*t*(9; 11)	0	0		0	0		0	0		1 (1.3)	1 (1.8)		1 (1.2)	6 (8.6)	
*t*(6; 9)	1 (0.3)	3 (0.9)		0	2 (0.7)		0	0		0	0		0	0	
Cytogenetic abnormalities = 2	0	0		0	0		0	0		0	0		16 (18.6)	8 (11.4)	
Complex abnormalities (>=3)	3 (1)	6 (1.8)		2 (0.6)	5 (1.6)		0	0		1 (1.3)	0		10 (11.6)	16 (22.9)	
Other	9 (2.9)	17 (5.2)		13 (4.1)	21 (6.9)		0	0		0	0		11 (12.8)	17 (24.3)	
Unknown	176 (57.5)	204 (62)		182 (57.6)	195 (63.9)		0	0		7 (9.3)	5 (8.8)		4 (4.7)	3 (4.3)	
OS															
Dead	155 (50.7)	273 (83)	***	166 (52.5)	240 (78.7)	***	48 (55.8)	55 (71.4)	*	38 (50.7)	42 (73.7)	**	30 (34.9)	46 (65.7)	***
Alive	151 (49.3)	56 (17)		150 (47.5)	65 (21.3)		38 (44.2)	22 (28.6)		37 (49.3)	15 (26.3)		56 (65.1)	24 (34.3)	

The chi‐square test was used in Table 1 except age, BM blast cells. The *t* test was used to test the difference between age, BM blast cells in two risk groups.

Abbreviations: BM, bone marrow; FAB, French‐American‐British classification systems; HB, haemoglobin; ns, no significant; PLT, platelet counts; WBC, white blood cell counts.

^a^ In training data set, 42 and 49 age information was unknown in the low‐risk and high‐risk groups, respectively.

^b^ In testing data set, 35 and 41 age information was unknown in the low‐risk and high‐risk groups, respectively.

^c^ The unit of age was days in TARGET data set. Other data sets were years.

^d^ In TCGA data set, 9 BM blast cell information was unknown in the both low‐risk and high‐risk groups.

^e^ In TARGET data set, 4 BM blast cell information was unknown in the high‐risk group.

* <.05.

** <.01.

*** <.001.

### 3.5 Multivariate analysis in two risk groups

**Figure 6:**
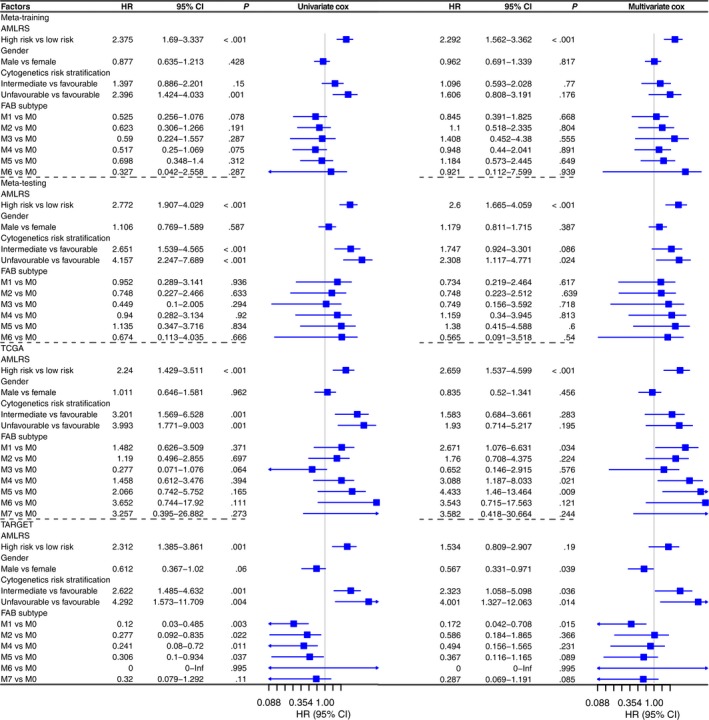
The univariate and multivariate COX regression analyses for risk stratification and clinical variables including gender, cytogenetic risk stratification and FAB subtype in four data sets. FAB, French‐American‐British

To further validate the prognostic power of 10‐gene signature, univariate and multivariate analyses based on COX regression without missing data were carried out for clinical variables and AMLRS in four data sets. Clinical variables that may be associated with prognosis were included in the analysis, including gender, cytogenetic risk stratification and FAB subtype. As can be seen from Figure 6, the AMLRS was illustrated to be a significantly independent prognostic factor in four data sets after elimination of the effects of confounding factor (meta‐training data set, HR 2.292, 95% CI 1.562‐3.362, *P* < .001; meta‐testing data set, HR 2.6, 95% CI 1.665‐4.059, *P* < .001; TCGA data set, HR 2.659, 95% CI 1.537‐4.599, *P* < .001; TARGET data set, HR 1.534, 95% CI 0.809‐2.907, *P* = .19). In Kaplan‐Meier survival analysis, the low‐risk group had longer OS than those in the high‐risk group in different cytogenetic risk stratification and M1, M2, M4 and M5 subtypes (*P* < .05; Figure S7). No statistically significant difference was found in M0, M3, M6 and M7 subtypes between the low‐risk and high‐risk groups, and this is possibly due to insufficient sample size.

### 3.6 Establishment of the predictive nomogram

**Figure 7:**
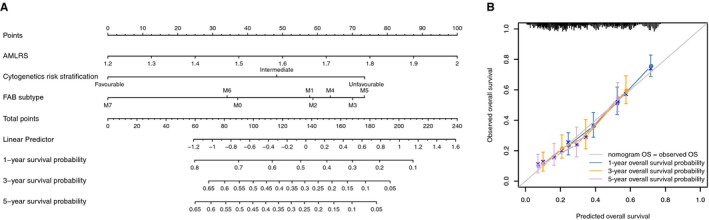
The nomogram to predict the probability of 1‐y, 3‐y and 5‐y OS in AML. (A) The nomogram was created incorporating with risk score, cytogenetic risk stratification and FAB subtype in all data sets. Calibration curve for observed and predicted 1‐y, 3‐y and 5‐y OS in all data (B). OS, overall survival; FAB, French‐American‐British

For the convenience of clinical application, a clinically quantitative method was expected to produce that could predict the probability of 1‐year, 3‐year and 5‐year OS in AML. Therefore, using data without missing data from meta‐training, meta‐testing, GPL96‐http://www.ncbi.nlm.nih.gov/geo/query/acc.cgi?acc=GSE12417, TCGA and TARGET data sets, we conducted a nomogram which merge AMLRS and two clinically correlated risk factors, including FAB subtype and cytogenetic risk stratification (Figure 7A). As can be seen in calibration plots for the 1‐year, 3‐year and 5‐year OS, the nomogram was predicted well in all data, and concordance index was 0.6542 (*P* = .0157; Figure 7B). In order to make clinical use convenient, we created an online tool predicting prognosis of AML patients (https://tcgi.shinyapps.io/amlrs_nomogram/).

## 4 DISCUSSION

With the flourish of precision medicine and the accumulation of clinical and biological data, increasing scholars have dedicated their effort to lucubrate the diagnostic or prognostic prediction models, and prediction research for AML patients is no exception.^16^, ^17^ There are already some published AML signatures, such as 17‐gene stemness score (LCS17), 3‐microRNA prognostic scoring system, 24‐gene prognostic signature and so on. LSC17 performed very well in predicting the prognosis of AML patients in different data sets. However, some clinically applied risk stratification, such as cytogenetic risk stratification, should continue to be used.^17^ A 3‐microRNA prognostic scoring system was constructed by Chuang et al, which applied private and public databases. However, the patient number in training and validation cohorts both is small, which reduced the stability and accuracy of the prognostic signature, and study population is only for adult AML patients.^18^ Another 24‐gene prognostic signature was established using private database by Li et al which comprised various cytogenetic and molecular abnormalities AML patients and was validated by two independent cohorts.^19^ Nevertheless, excessive gene number being used to constructed prognostic signature might produce adverse effects of model overfitting. Besides, Zhu et al generated a 4‐microRNA prognostic signature in paediatric and adolescent AML patients.^20^ Unfortunately, because of shortage of other microRNA with survival information, the model was validated only in the testing data set, but not in independent data set.

In our study, we downloaded almost available multiple gene expression data and corresponding clinical information from the GEO, TCGA and TARGET database, and merged data from different platform after eliminating batch effects. In meta‐training data set, screened by univariate COX, log‐rank test and LASSO‐COX method, 10 genes were ultimately identified to create AMLRS. Subsequently, the AMLRS was validated by meta‐testing data set and three independent validation data sets. The result indicated that AMLRS could divide well AML patients into two instinct subgroups with the low‐risk and high‐risk groups. Meanwhile, in order to eliminate effects of farraginous factors, the AMLRS and clinical parameters were analysed by univariate and multivariate COX regression without missing data. The AMLRS involved in 10 prognostic genes was proven to have independent prognostic value in AML patients.

Compared with previous studies, our study not only combines data from multiple platforms by Combat method, which expanded the sample size to a large extent, but also incorporates different clinical parameters. Meanwhile, because overfitting could reduce significance of the prognostic signature, we adopted LASSO‐COX method for shrinkage and filtration of genes. In the selection of research object, our study has greater inclusiveness, including patients of different ages and different cytogenetic and molecular abnormalities. Based on these elements, a robust and reliable 10 gene prognostic signature of AML was accurately created for different types of AML patients.

Ten genes involved in the AMLRS have been investigated in our research, including ALDH2, FAM124B, MYNRIN, DNMT3B, DDIT4, SOCS2, ADGRG1, CALCRL, NDST1 and THL1. ALDH enzyme activity in haematopoietic system is utilized to define normal haematopoietic stem cell, but previous research has shown that ALDH activity might be related to the existence of leukaemic stem cells, and its high activity might be a reminder of poor prognosis.^21^ DNMT3B, as a member of DNA methyltransferases family proteins, functions chiefly as de novo DNA methyltransferases to create new DNA methylation marks.^22^, ^23^ Overexpression of DNMT3B impaired leukaemogenesis and postponed the progress of leukaemia.^24^ Nevertheless, several recent researches of DNMT3B indicated that high expression of DNMT3B was connected with unfavourable outcome in AML.^24^, ^25^ In general, DNMT3B‐mediated DNA methylation plays an important role in the onset and progression of AML. DDIT4 was mainly applied to restrain mechanistic target of rapamycin 1 (mTORC1) by maintaining the TSC1‐TSC2 inhibitory complex.^26^ The up‐regulation of DDIT4 has been reported as prognostic biomarker in AML.^27^, ^28^ SOCS2 was expressed in both normal HSC and AML‐LSC,^29^ and did its job by inhibiting the JAK/STAT pathway.^30^ Overexpression of SOCS2 was mostly associated with the advanced stages of chronic myeloid leukaemia.^31^, ^32^, ^33^ However, there were also studies showing that high expression of SOCS2 in paediatric AML patients had an inferior prognosis.^34^ Although not reported to be associated with the prognosis of AML patients, ADGRG1, NDST1, FHL1, FAM124B, NYNRIN and CALCRL had been reported to be related with several other cancers 35, 36, 37 and might be potential novel prognostic factors of AML.

In addition, for the sake of the facility of clinical application, a nomogram was produced including AMLRS, cytogenetic risk stratification and FAB subtype. In order to make clinical use convenient, we created an online tool predicting prognosis of AML patients (https://tcgi.shinyapps.io/amlrs_nomogram/). In all data, the nomogram performed well.

However, there is certain deficiency in our study. First of all, this was a retrospective research based on public database. The missing rate of clinical information data was comparatively high, and the white race was in the majority, which lowered the stability and reliability of the prognostic signature. There were M3 subtypes in research cohort, the prognosis of which was very different. But owing to a small amount of these patients, we did not analyse it separately, which also caused a certain deviation in the prediction effect of the model. Furthermore, gene for screening was the gene in intersection after merging data from various databases. Certain genes with less expression and outside the intersection were missed, which led to error. Last but not least, because of insufficiency of clinical parameters in our study, the end‐point was only OS, which brought about the missing of clinical parameters that might have an impact on the outcome. For the next step, we will take AML patients in our centre as the research object to expand the proportion of Asians and incorporate more clinical parameters for analysis, as well as set additional end‐point to observe the outcome of different events. Meantime, we will develop a gene quantification batch effect elimination tool that allows individual patients to use gene expression quantification for risk stratification. We, if available, will explore the possible mechanism of prognostic genes.

## 5 CONCLUSIONS

In conclusion, a promising prognostic signature based on 10 genes related to the prognosis was recognized for prognostic risk stratification of AML. Meanwhile, a nomogram and an online tool were built to easy to clinical application. However, the relevant mechanisms of the probable prognosis genes have not been distinctly identified, perhaps these genes will become potential therapeutic target in future.

## CONFLICT OF INTEREST

The authors confirm that there are no conflicts of interest.

## AUTHOR CONTRIBUTION

ZY, JS and XC designed the study. JS, NL and LZ collected the data. ZY and JS performed statistical analyses. ZY, TT and GT did literature research. ZY wrote the manuscript. All authors read and approved the final manuscript.

## Supporting information

Supplementary Material

## Data Availability

The data sets of this study were generated from the TCGA database, the GEO database and the TARGET database.
