# Supplementary material for: Development and validation of a 10‐gene prognostic signature for acute myeloid leukaemia
Source: J Cell Mol Med. 2020 Mar 9;24(8):4510–23. doi: 10.1111/jcmm.15109 (PMC7176885; doi:10.1111/jcmm.15109)
Supplement: Supplementary file 1 — Supplementary Material [file JCMM-24-4510-s001.pdf]

Supplementary: Development and validation of a 10-gene prognostic signature for acute myeloid leukemia

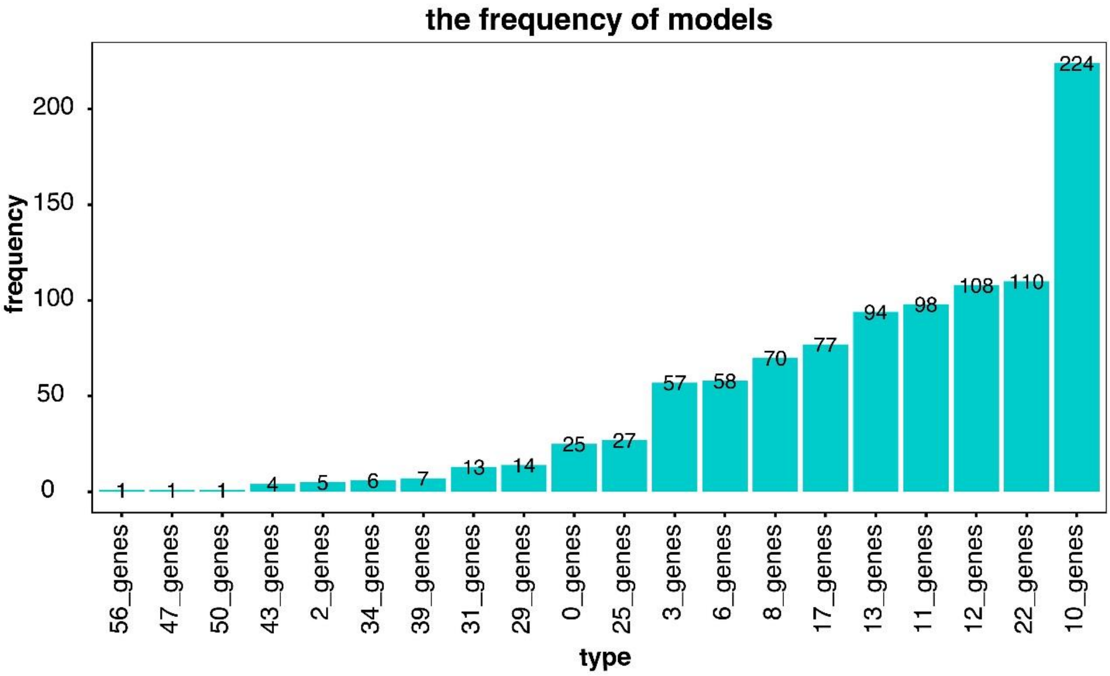

Figure S1 The frequency distribution of signature models

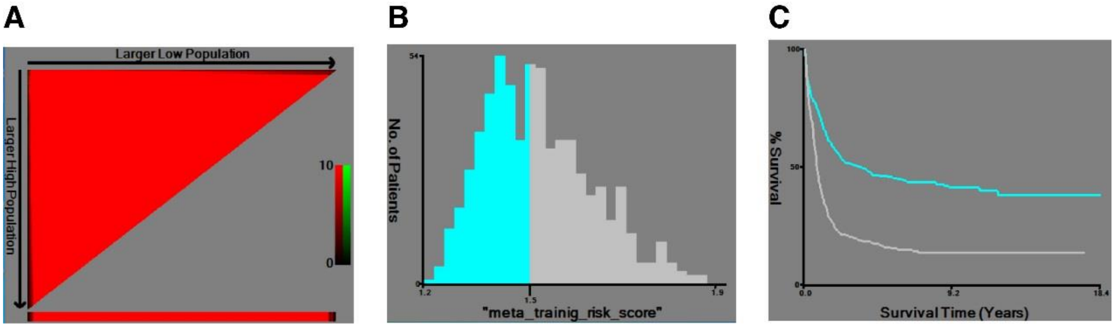

Figure S2 The optimal cutoff value (cutoff = 1.47) of AMLRS was generated by X-tile in training cohort.

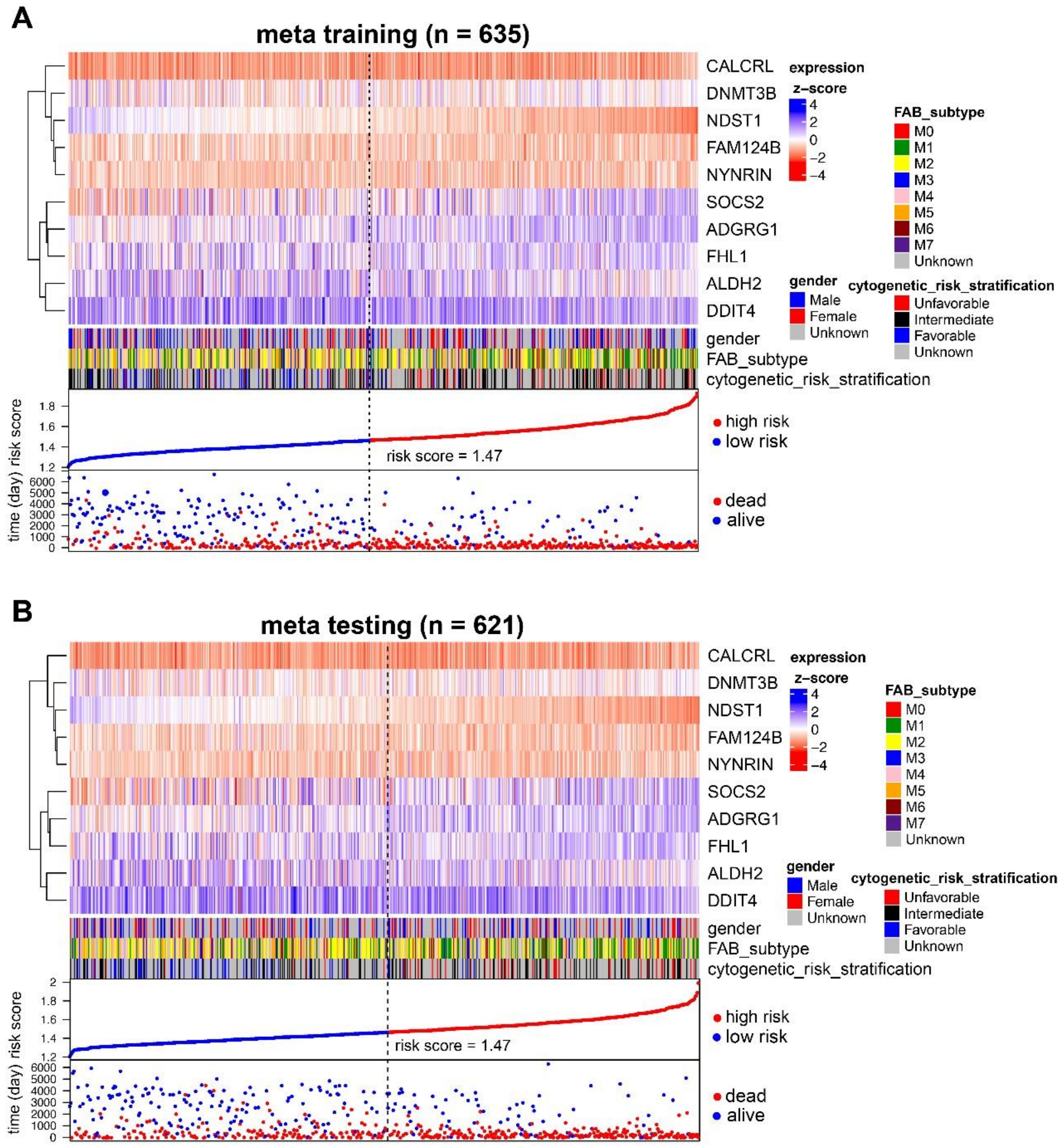

Figure S3 The heat map of risk score distribution of 10 survival-related genes and clinical characteristics in meta training and meta testing datasets.
(A) meat training dataset; (B) meta testing dataset.

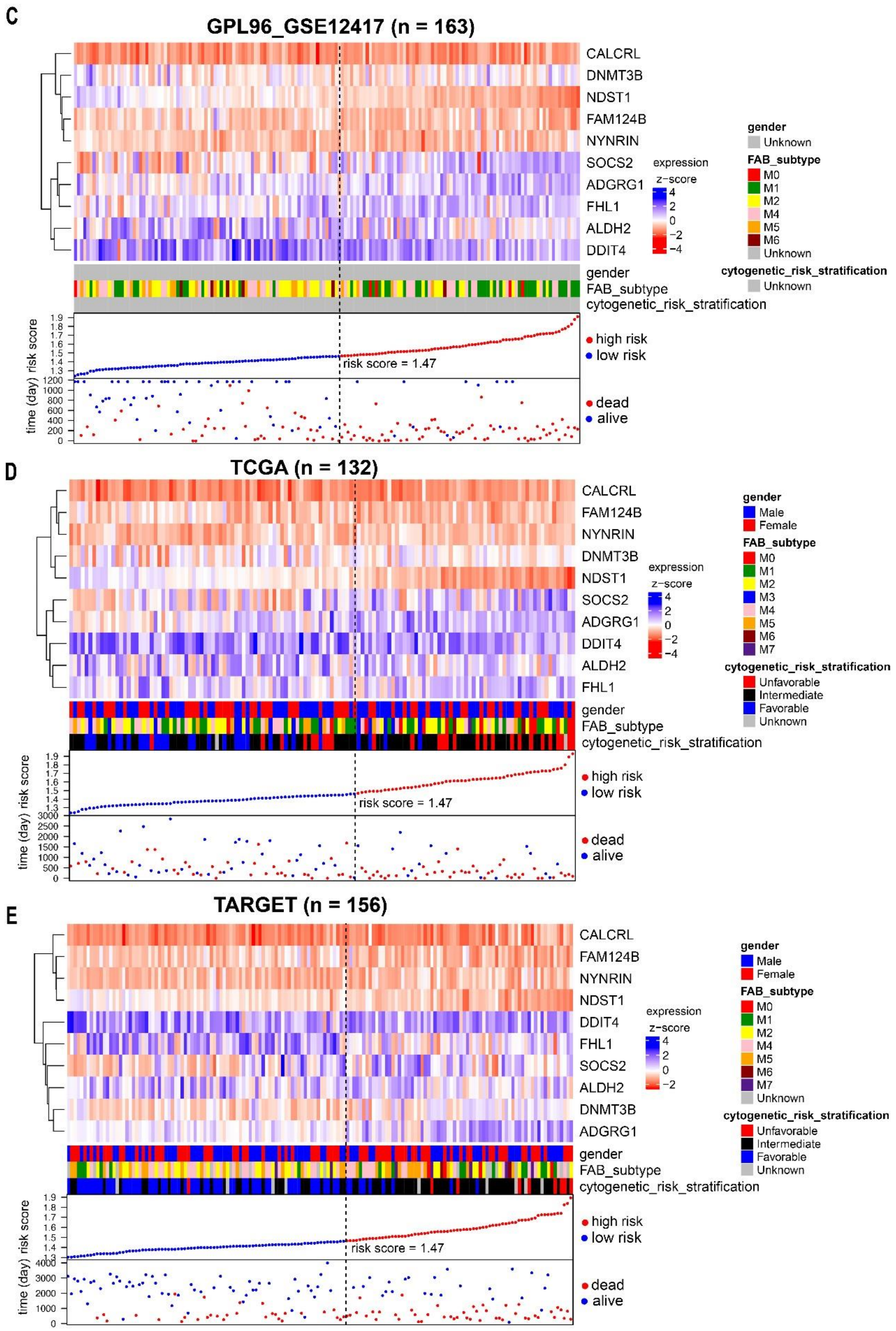

Figure S3 The heat map of risk score distribution of 10 survival-related genes and clinical characteristics in independent validation cohorts.
(C) GPL96_GSE12417 dataset; (D) TCGA dataset; (E) TARGET dataset.

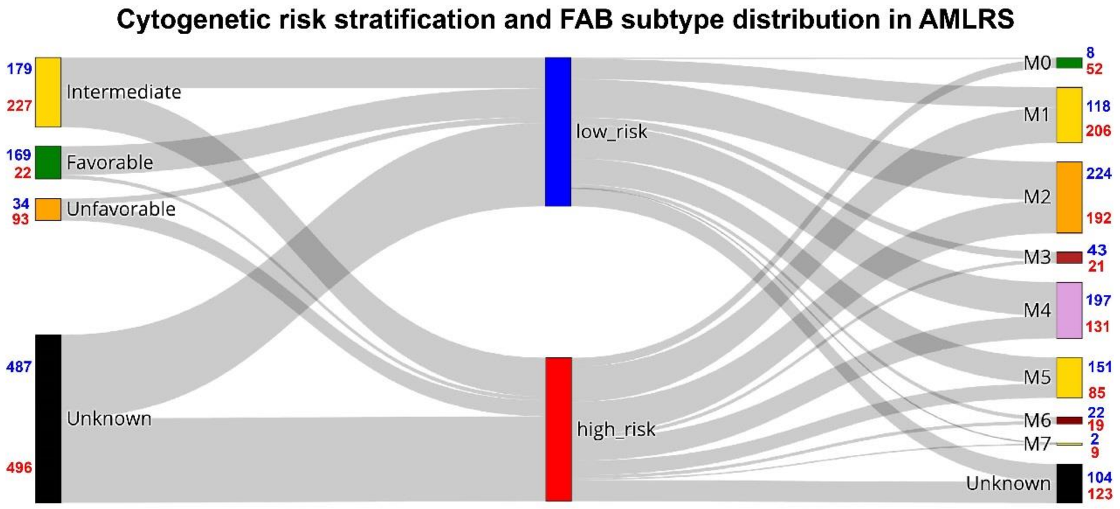

Figure S4 The distribution of cytogenetic risk stratification and FAB classification in low risk and high risk group. The red number represents the number of people in the high-risk group, and the blue number represents the number of people in the low-risk group.

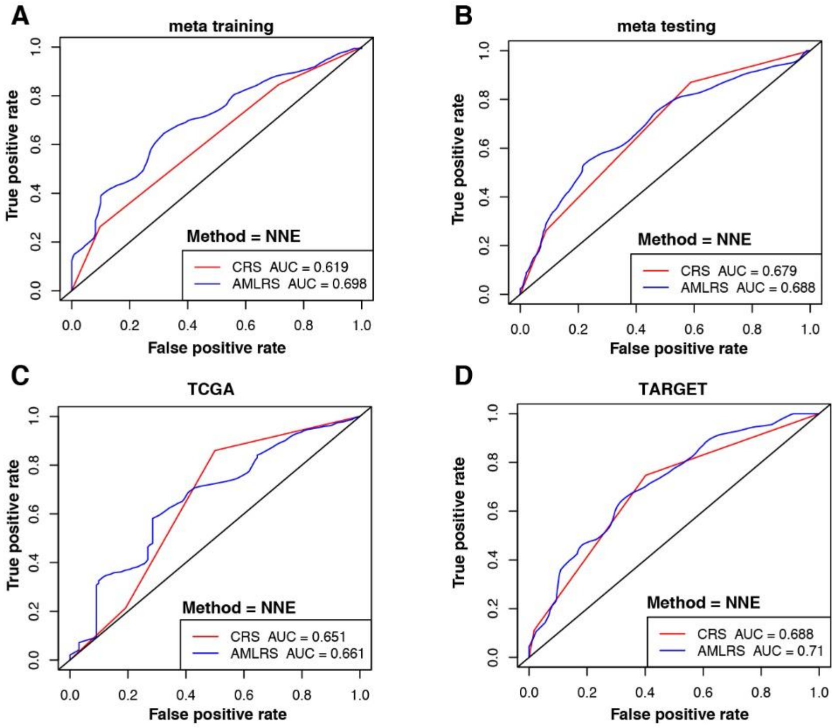

Figure S5 The 5-year time-dependent ROC of cytogenetic risk stratification (CRS) and AMLRS in 5 datasets. (A) In meta training dataset the 5-year AUC of CRS and AMLRS are 0.619 and 0.698, respectively; (B) In meta testing dataset, the 5- year AUC of CRS and AMLRS are 0.679 and 0.688, respectively; (C) In TCGA dataset, the 5-year AUC of CRS and AMLRS are 0.651 and 0.661, respectively;(D) In TARGET dataset, the 5-year AUC of CRS and AMLRS are 0.688 and 0.71, respectively

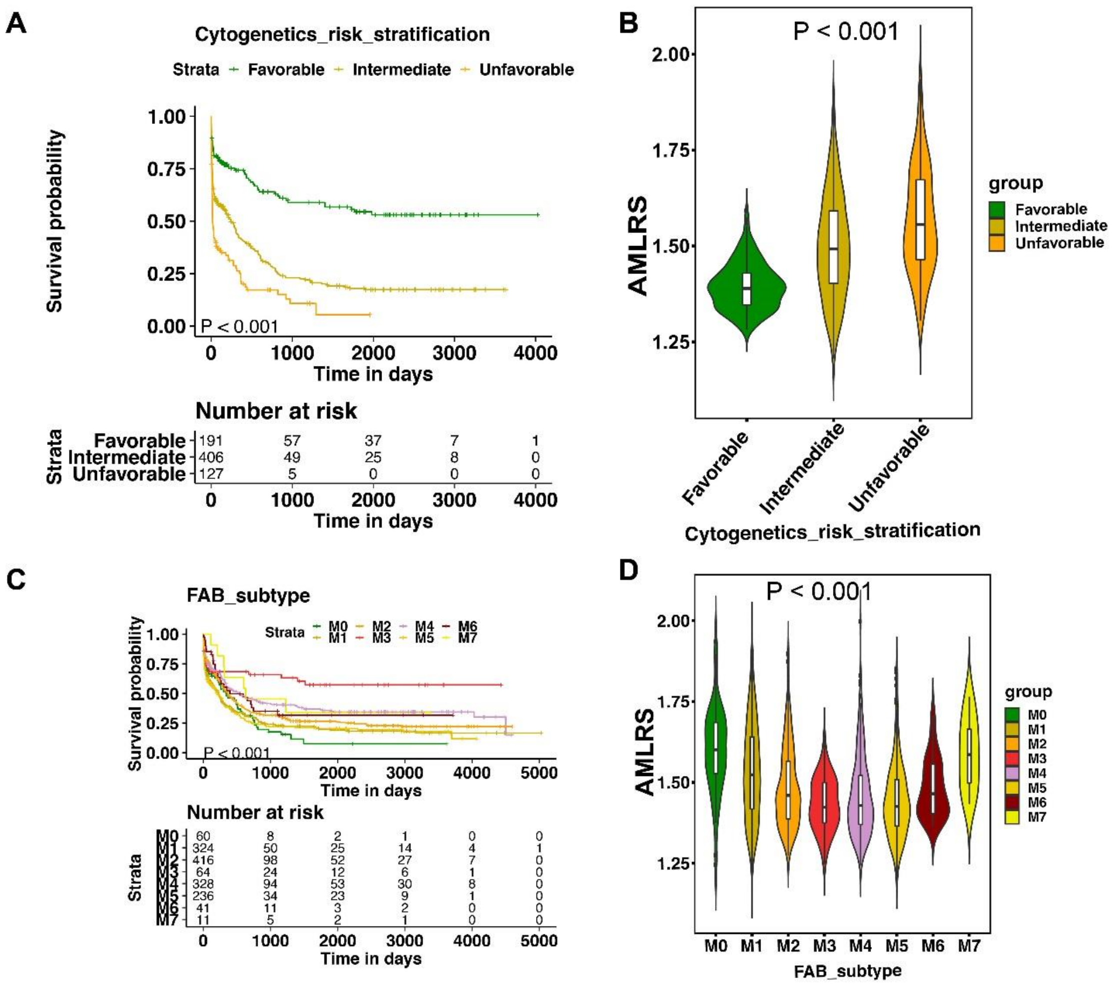

Figure S6 The subgroup analysis of cytogenetic risk stratification and FAB classification. The Kaplan-Meier survival analysis of cytogenetic risk stratification (A) and FAB classification (C). The boxplot of cytogenetic risk stratification (B) and FAB classification (D).

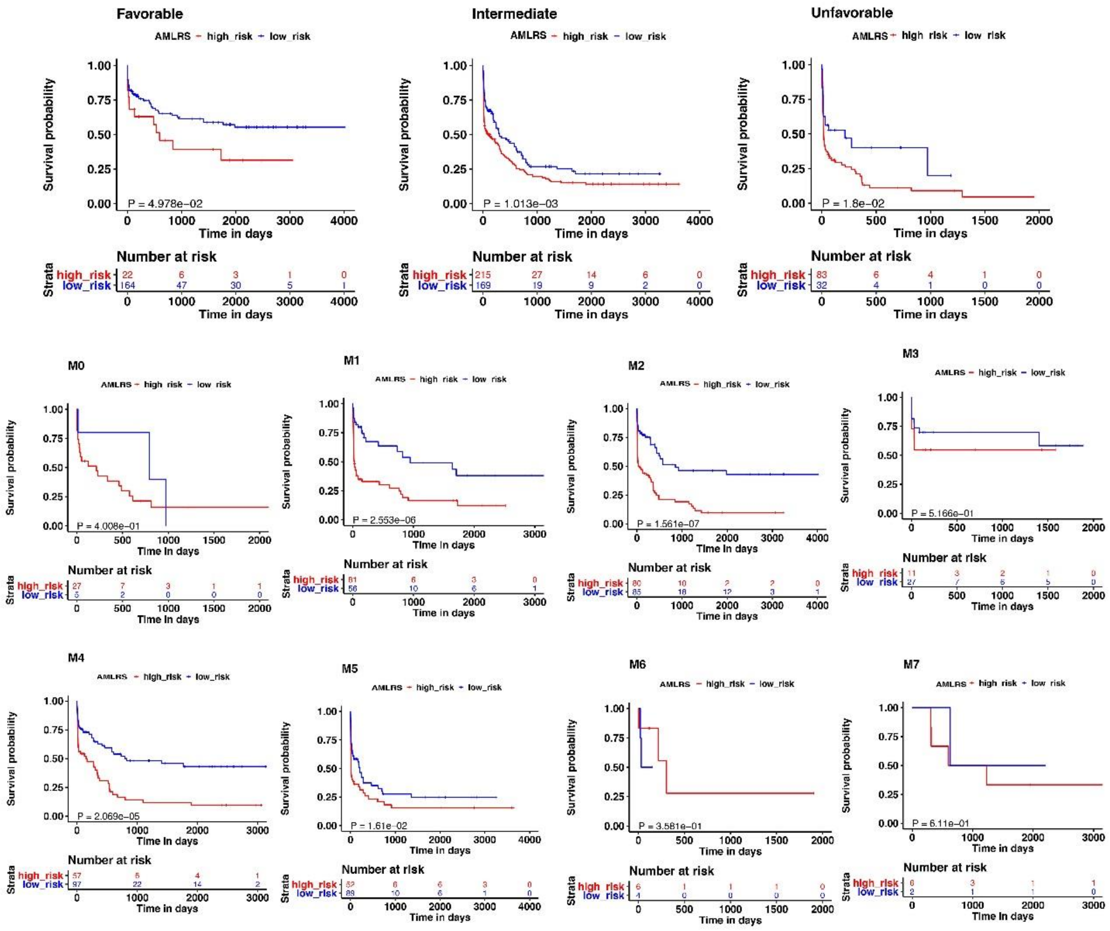

Figure S7 The Kaplan-Meier survival analysis of cytogenetic risk stratification and FAB classification between low risk and high risk group.

TableS1: Detail information about the datasets in this project

Dataset	Dataset	Caner type	Platform	Samples
Meta (training/testing) dataset	GSE6891	AML	[HG-U133_Plus_2] Affymetrix Human Genome U133 Plus 2.0 Array	520
	GSE12417	AML	[HG-U133_Plus_2] Affymetrix Human Genome U133 Plus 2.0 Array	79
	GSE37642	AML	[HG-U133A] Affymetrix Human Genome U133A Array	417
	GSE37642	AML	[HG-U133_Plus_2] Affymetrix Human Genome U133 Plus 2.0 Array	136
	GSE71014	AML	Illumina HumanHT-12 V4.0 expression beadchip	104
Independent Validation	GSE12417	AML	[HG-U133A] Affymetrix Human Genome U133A Array	163
	TARGET	AML	Illumina HiSeq	156
	TCGA	AML	Illumina HiSeq	132

Table S2 Clinical characteristics of AML patients in 5 datasets.

Clinical factors	Meta training (n = 635)	Meta testing (n = 621)	GPL96_GSE12417 (n = 163)	TCGA (n = 132)	TARGET (n = 156)	Total (n = 1707)
**Gender**						
Male	127	113	0	71	79	390 (22.8%)
Female	115	102	0	61	77	355 (20.8%)
Unknown	393	406	163	0	0	962 (56.4%)
**Age at diagnosis**	50(15-83)	53(15-85)	58(17-83)	55(21-88)	9.05(0.38-22.55)	47(0.38-88)
**Race**						
White	0	0	0	118	121	239 (14.0%)
Black or African American	0	0	0	11	16	27 (1.6%)
Asian	0	0	0	1	3	4 (0.2%)
Other	0	0	0	0	10	10 (0.6%)
Unknown	635	0	163	2	6	1427 (83.6%)
**Initial blood cell counts**						
White blood cell counts						
<4	0	0	0	30	7	37 (2.2%)
4-10	0	0	0	23	13	36 (2.1%)
>10	0	0	0	78	136	214 (12.5%)
None	0	0	0	1	0	1420 (83.2%)
Hemoglobin						
<12	0	0	0	118	0	118 (6.9%)
≥12	0	0	0	13	0	13 (0.8%)
None	0	0	0	1	0	1576 (92.3%)
Platelet counts						
<100	0	0	0	106	0	106 (6.2%)
≥100	0	0	0	26	0	26 (1.5%)
**Bone marrow blasts (%)**	0	0	0	45(2-97)	74.3(14-100)	1575 (92.3%)
**FAB subtype**						
M0	19	20	5	12	4	60 (3.5%)
M1	117	113	45	32	17	324 (19.0%)
M2	148	154	45	32	37	416 (24.4%)
M3	26	24	0	14	0	64 (3.7%)
M4	107	109	42	27	43	328 (19.2%)
M5	91	84	19	12	30	236 (13.8%)
M6	14	17	6	2	2	41 (2.4%)
M7	2	1	0	1	7	11 (0.6%)
Unknown	111	99	1	0	16	227 (13.3%)
**Cytogenetic risk stratification**						
Favorable	46	51	0	30	64	191 (11.2%)
Intermediate	140	119	0	73	74	406 (23.8%)
Unfavorable	50	41	0	27	9	127 (7.4%)
Unknown	399	410	163	2	9	983 (57.6%)
**Cytogenetic abnormality**						
Normal	141	125	163	62	35	526 (30.8%)
-5/7 (q)	16	14	0	16	0	46 (2.7%)
+8	11	9	0	11	3	34 (2.0%)

11q23	5	5	0	0	0	10 (0.6%)
inv(16)	17	16	0	8	21	62 (3.6%)
t(9;22)	1	1	0	0	0	2 (0.1%)
t(15;17)	9	12	0	13	0	34 (2.0%)
t(8;21)	16	19	0	7	5	47 (2.8%)
t(9;11)	0	0	0	2	7	9 (0.5%)
t(6;9)	4	2	0	0	0	6 (0.4%)
Cytogenetic abnormalities=2	0	0	0	0	24	24 (1.4%)
Complex abnormalities (>=3)	9	7	0	1	26	43 (2.5%)
Other	26	34	0	0	28	88 (5.2%)
Unknown	380	377	0	12	7	776 (45.5%)
**OS**						
Dead	428	406	103	80	76	1093 (64.0%)
Alive	207	215	60	52	80	614 (36.0%)

Table S3 The frequency of models and detailed information of genes.

type	genes	frequency
29_genes	ALDH2;FAM124B;PLSCR4;HSPB1;SPINT2;NYNRIN;QRICH1;CLIC4P1;DNMT3B;ADRA2A;POM121;DDIT4;TDRKH;SOCS2;CNOT8;ADGRG1;FRYL;ACP6;TRPS1;CALCRL;NDST1;SQLE;KIAA0232;POLDIP3;FHL1;FADS1;YKT6;LAPTM4B;ZNF74	14
12_genes	ALDH2;FAM124B;NYNRIN;DNMT3B;DDIT4;SOCS2;ADGRG1;CALCRL;NDST1;FHL1;LAPTM4B;ZNF74	108
10_genes	ALDH2;FAM124B;NYNRIN;DNMT3B;DDIT4;SOCS2;ADGRG1;CALCRL;NDST1;FHL1	224
17_genes	ALDH2;FAM124B;PLSCR4;NYNRIN;CLIC4P1;DNMT3B;POM121;DDIT4;TDRKH;SOCS2;ADGRG1;CALCRL;NDST1;FHL1;FADS1;LAPTM4B;ZNF74	77
25_genes	ALDH2;FAM124B;PLSCR4;HSPB1;NYNRIN;QRICH1;CLIC4P1;DNMT3B;POM121;DDIT4;TDRKH;SOCS2;ADGRG1;FRYL;TRPS1;CALCRL;NDST1;SQLE;KIAA0232;POLDIP3;FHL1;FADS1;YKT6;LAPTM4B;ZNF74	27
6_genes	DNMT3B;DDIT4;SOCS2;ADGRG1;CALCRL;FHL1	58
11_genes	ALDH2;FAM124B;NYNRIN;DNMT3B;DDIT4;SOCS2;ADGRG1;CALCRL;NDST1;FHL1;LAPTM4B	98
22_genes	ALDH2;FAM124B;PLSCR4;HSPB1;NYNRIN;QRICH1;CLIC4P1;DNMT3B;POM121;DDIT4;TDRKH;SOCS2;ADGRG1;CALCRL;NDST1;SQLE;KIAA0232;POLDIP3;FHL1;FADS1;LAPTM4B;ZNF74	110
0_genes		25
13_genes	ALDH2;FAM124B;PLSCR4;NYNRIN;DNMT3B;DDIT4;SOCS2;ADGRG1;CALCRL;NDST1;FHL1;LAPTM4B;ZNF74	94
43_genes	ALDH2;ADCY6;CAVIN3;HCP5;FAM124B;PLSCR4;HSPB1;SPINT2;QRICH1;CLIC4P1;DNMT3B;ADRA2A;POM121;DDIT4;LSP1;TDRKH;SOCS2;KIF26B;TNNT1;CNOT8;CACNA1B;ADGRG1;GAS6;SENP2;CRTAC1;FRYL;ACP6;GDE1;TRPS1;CALCRL;TMEM144;CAMK4;MELTF;CYP2E1;NDST1;SQLE;KIAA0232;POLDIP3;FHL1;FADS1;YKT6;LAPTM4B;ZNF74	4
2_genes	ADGRG1;CALCRL	5
8_genes	FAM124B;NYNRIN;DNMT3B;DDIT4;SOCS2;ADGRG1;CALCRL;FHL1	70
3_genes	SOCS2;ADGRG1;CALCRL	57
34_genes	ALDH2;FAM124B;PLSCR4;HSPB1;SPINT2;QRICH1;CLIC4P1;DNMT3B;ADRA2A;POM121;DDIT4;LSP1;TDRKH;SOCS2;KIF26B;TNNT1;CNOT8;CACNA1B;ADGRG1;GAS6;FRYL;ACP6;GDE1;TRPS1;CALCRL;NDST1;SQLE;KIAA0232;POLDIP3;FHL1;FADS1;YKT6;LAPTM4B;ZNF74	6
31_genes	ALDH2;FAM124B;PLSCR4;HSPB1;SPINT2;NYNRIN;QRICH1;CLIC4P1;DNMT3B;ADRA2A;POM121;DDIT4;TDRKH;SOCS2;KIF26B;CNOT8;ADGRG1;FRYL;ACP6;GDE1;TRPS1;CALCRL;NDST1;SQLE;KIAA0232;POLDIP3;FHL1;FADS1;YKT6;LAPTM4B;ZNF74	13
39_genes	ALDH2;CAVIN3;FAM124B;PLSCR4;HSPB1;SPINT2;QRICH1;CLIC4P1;DNMT3B;ADRA2A;POM121;DDIT4;LSP1;TDRKH;SOCS2;KIF26B;TNNT1;CNOT8;CACNA1B;ADGRG1;GAS6;CRTAC1;FRYL;ACP6;GDE1;TRPS1;CALCRL;TMEM144;CAMK4;MELTF;NDST1;SQLE;KIAA0232;POLDIP3;FHL1;FADS1;YKT6;LAPTM4B;ZNF74	7
56_genes	ULK1;ALDH2;ADCY6;CAVIN3;NKRF;CUZD1;HCP5;FAM124B;PLSCR4;HSPB1;CXCL3;SPINT2;QRICH1;FXYD1;CLIC4P1;PTPRN;DNMT3B;ADRA2A;POM121;DDIT4;LSP1;HYAL1;TDRKH;SOCS2;KIF26B;ARHGAP32;TNNT1;CNOT8;RNASET2;CACNA1B;ARNTL2;ADGRG1;ZAP7	1

	0;GAS6;SENP2;ZMAT3;CRTAC1;FRYL;GDE1;TRPS1;CALCRL;TMEM144;BAG4;CAMK4;MELTF;CYP2E1;IL1R1;ARFGEF2;NDST1;SQLE;KIAA0232;POLDIP3;FADS1;DDA1;LAPTM4B;ZNF74	
47_genes	ALDH2;ADCY6;CAVIN3;HCP5;FAM124B;PLSCR4;HSPB1;SPINT2;QRICH1;FXYD1;CLIC4P1;DNMT3B;ADRA2A;POM121;DDIT4;LSP1;HYAL1;TDRKH;SOCS2;KIF26B;ARHGAP32;TNNT1;CNOT8;CACNA1B;ADGRG1;ZAP70;GAS6;SENP2;CRTAC1;FRYL;ACP6;GDE1;TRPS1;CALCRL;TMEM144;CAMK4;MELTF;CYP2E1;NDST1;SQLE;KIAA0232;POLDIP3;FHL1;FADS1;DDA1;LAPTM4B;ZNF74	1
50_genes	ALDH2;ADCY6;CAVIN3;HCP5;FAM124B;PLSCR4;HSPB1;SPINT2;QRICH1;FXYD1;CLIC4P1;DNMT3B;ADRA2A;POM121;DDIT4;LSP1;HYAL1;TDRKH;SOCS2;KIF26B;ARHGAP32;TNNT1;CNOT8;CACNA1B;ARNTL2;ADGRG1;ZAP70;GAS6;SENP2;CRTAC1;FRYL;ACP6;GDE1;TRPS1;CALCRL;TMEM144;BAG4;CAMK4;MELTF;CYP2E1;ARFGEF2;NDST1;SQLE;KIAA0232;POLDIP3;FHL1;FADS1;DDA1;LAPTM4B;ZNF74	1
